# On Albumen in the Urine of Various Diseases

**Published:** 1853-03

**Authors:** 


*On Albumen in the Urine of Various Diseases.*—Heller asserts that albumen is present in the urine in all kidney-lesions, though sometimes in small quantity, and that it exists in many other diseases, and often in greater amount.

1. *Pneumonia* and *Tuberculosis acuta.*—At the commencement of exudation, while yet the chloride in the urine is in undiminished quantity, no albumen can be found. As exudation increases, and as the chloride in the urine diminishes, a very small quantity of albumen appears, and continues for a long time. This appearance is not constant, but is very frequent. The greater the albumen, and the less the chloride in the urine, so much the worse is the prognosis.

2. *Pleurisy.*—Albumen does not appear so frequently, even when the chloride is much diminished. In the period of absorption it sometimes occurs, and is attended with carbonate and hydrothionate of ammonia.

3. *Acute Liver-Affections.*—In chronic or subacute inflammations, where the chloride of the urine is diminished, albumen appears as in pneumonia.

4. *Pericarditis* and *Endocarditis.*—In the first case albumen sometimes occurs; in the last, very seldom even when the chloride is much diminished.

5. *Peritonitis.*—Albumen is frequently found, and continues sometimes long after the customary amount of chloride has reappeared, and morbus Brightii is then, perhaps, left.

6. *Metritis* and *Eclampsia Puerperalis.*—As in peritonitis.

7. *Cholera.*—More or less albumen.—*Prov. Jour, from Archiv. für Pathol. Chem.*, Band i. Heft 8.

